# Organ Tropism of *Angiostrongylus vasorum* Larval Stages in Infected African Giant Snails (*Lissachatina fulica*)

**DOI:** 10.3390/pathogens13110946

**Published:** 2024-10-30

**Authors:** Alena Dusch, Lisa Segeritz, Manfred Henrich, Anja Taubert, Carlos Hermosilla

**Affiliations:** 1 Institute of Parasitology, Biomedical Research Center Seltersberg (BFS), Justus Liebig University Giessen, 35392 Giessen, Germany; lisa.segeritz@gmail.com (L.S.); anja.taubert@vetmed.uni-giessen.de (A.T.); carlos.r.hermosilla@vetmed.uni-giessen.de (C.H.); 2 Institute of Pathology, Justus Liebig University Giessen, 35392 Giessen, Germany; manfred.henrich@vetmed.uni-giessen.de

**Keywords:** *Angiostrongylus vasorum*, *Lissachatina fulica*, innate immunity, hemocytes, organ tropism

## Abstract

*Angiostrongylus vasorum* is a metastrongyloid lungworm causing severe cardiovascular disease in domestic and wild animals. During its heteroxenous life cycle, *A. vasorum* requires obligate gastropod intermediate hosts. Little is known about *A. vasorum* larval organ tropism and development in gastropod intermediate hosts. Thus, the aim of this study was to analyze in vivo development of *A. vasorum* larval stages in experimentally infected African giant snails (*Lissachatina fulica*). Adult *L. fulica* (*n* = 26) were orally infected with *A. vasorum*-L1 and thereafter continuously euthanized. Gastropod organs were artificially digested and microscopically analyzed for the presence of *A. vasorum* larvae. Moreover, paraffin-fixed organs were investigated histologically for snail-borne innate immune response. In the current study, the success of *L. fulica* oral infection was demonstrated, thereby reaching larval recovery rates of up to 49.7%. During snail infection, an organ tropism of *A. vasorum* larvae was detected for the lungs and the foot muscular tissue. Overall, *A. vasorum*-driven gastropod innate immune reactions against larvae varied greatly. In some specimens, larvae were found effectively ensnared by recruited hemocytes, resulting in granuloma formation, whilst in others, hemocyte-mediated reactions were barely observed. Nevertheless, these evidences demand more studies on hemocyte-derived effector mechanisms against *A. vasorum*.

## 1. Introduction

*Angiostrongylus vasorum* (Baillet, 1866) is a metastrongyloid lungworm that resides in the cardiovascular system of both domestic and wild canids, including dogs (*Canis familiaris*), wolves (*Canis lupus*) [1,2], golden jackals (*Canis aureus*) [3,4], and red foxes (*Vulpes vulpes*) [5,6,7,8,9], with the latter representing important wildlife reservoirs in Europe. Further definitive hosts include striped skunks (*Mephitis mephitis*), weasels (*Mustela erminea*), ferrets (*Mustela putorius furo*), badgers (*Meles meles*), neotropical otters (*Lontra longicaudis*), raccoon dogs (*Nyctereutes procyonoides*), and red pandas (*Ailurus fulgens*) [10,11,12,13,14]. Due to the rising urbanization of many of these wild animals, the risk of *A. vasorum* transmission to domestic dogs and vice versa is increasing [1,15]. This metastrongyloid parasite can be found on multiple continents and generally shows a patchy epizootiological distribution, with hyper- and hypoendemic foci in close proximity [16,17]. Clinical signs of *A. vasorum* infections in dogs may vary from mild respiratory symptoms to severe cardiopulmonary disorders but also include hemorrhage and neurological, ocular, and gastrointestinal symptoms [13,18,19,20,21,22].

The life cycle of *A. vasorum* is heteroxenous, with terrestrial gastropods acting as obligatory intermediate hosts. Thus, canine angiostrongylosis is considered a gastropod-borne parasitosis with a complex epizootiology, including various slug, semi-slug, and snail species as suitable intermediate hosts [15,23,24]. By feeding on the feces of an *A. vasorum*-infected definitive host, gastropod intermediate hosts become infected with first-stage larvae (L1). Another possibility is the active penetration of the gastropod epidermis by exogenous L1 [25,26]. While migrating in the mollusc intermediate hosts, *A. vasorum* L1 must molt into second-stage (L2) and third-stage larvae (L3). The canid definitive host will become infected by consuming either an *A. vasorum*-infected intermediate host carrying L3 or by a paratenic host (PH; e.g., amphibians, birds) hosting infectious L3. Alternatively, spontaneous shedding of *A. vasorum* L3 from dead intermediate hosts can occur, which are capable of surviving in the environment for some days or weeks, depending on the climatic condition [27]. As already stated, a wide spectrum of gastropod species may act as intermediate hosts, including the terrestrial African giant snail (*Lissachatina fulica*) (Bowdich, 1822) [28,29,30].

This gastropod species is considered one of the largest terrestrial snails, and its shell is cone-formed with an average length of 90 mm and contains a minimum of seven whorls in fully grown snails. The shell is medium brown and decorated with darker and lighter stripes, and the tip is usually lighter in color [31,32]. Originally native to East Africa, this terrestrial giant snail is now considered an invasive species (i.e., neozoa) in multiple tropical and subtropical countries, such as Colombia, the United States of America, Brazil, Ecuador, Indonesia, and India [11,33,34]. Furthermore, *L. fulica* has been identified as a natural intermediate host not only for *A. vasorum* but also for *Aelurostrongylus abstrusus*, *Crenosoma vulpis*, and *Troglostrongylus brevior* in the tropics of South America, thereby representing a risk factor for metastrongyloid transmission for both domestic and wild canids/felids [28]. Based on both the invasive nature of *L. fulica* and the emergence of canine angiostrongylosis as a cardiopulmonary disease in various continents, it is of great interest to gain further knowledge on *A. vasorum* L1 migration and organ tropism in *L. fulica* [35], which currently is still not fully understood. Several studies have shown that, after cutaneous or oral gastropod infection, *A. vasorum* L1 must migrate inside the mollusc to find adequate organs for fulfilling further development into L2 and, finally, infective L3 [36,37,38]. During this larval migration in vivo, *A. vasorum* larvae will be exposed to the gastropod host innate immune system, mainly composed of hemocytes present in the hemolymph, which might not only influence migration routes but also hamper larval development. As opposed to mammals, which own both an innate and an adaptive immune system, gastropods exclusively possess an innate immune system. Hematopoiesis in molluscs is complex and less studied when compared to other metazoan species [39]. Gastropod hematopoiesis leads to the generation of hemocytes (syn. amoebocytes) in hemolymph circulation, tissues, and organs [40,41]. Gastropod hemocytes constitute the main cellular component of hemolymph but, in addition, serve as resident cells at other sites, such as connective and vascular tissues [39,42,43]. Invertebrate hemocytes have multiple functions, such as the transport of molecules, wound repair, coagulation, and defense against invasive pathogens [41]. Hence, hemocytes show diverse effector mechanisms to fight invasive pathogens, such as phagocytosis [44], cell-mediated cytotoxicity, production of reactive oxygen species (ROS), and encapsulation [45]. More recently, also the release of gastropod invertebrate extracellular phagocyte traps (InEPT) against metastrongyloid larvae, including *A. vasorum*, has been reported, which is an effective immune response against invading L1 [40,41,46].

While several histological studies on hemocyte-derived innate immune reactions against nematode larvae exist, much less is known on metastrongyloid lungworm organ tropism [36,47,48], especially on gastropod intermediate hosts being experimentally infected under standardized laboratory conditions, including humidity, light, and temperatures mimicking natural field conditions [36,49]. The aim of this study was, therefore, to gain knowledge on *A. vasorum* larval migration in the gastropod intermediate host *L. fulica,* including organ tropism, with selected histological analyses to tackle hemocyte-mediated innate responses against invasive larvae in vivo.

## 2. Material and Methods

### 2.1. Gastropod Maintenance Under Standardized and Parasite-Free Conditions

German-bred African giant snails (*L. fulica*) were kept in a full-automatized climate chamber (ECP01E, Snijders Scientific B.V. Tilburg, the Netherlands) under standardized laboratory conditions with humidity and temperatures mimicking the gastropod’s natural habitat by temperatures ranging from 20 to 26 °C and 50% humidity, according to Penagos-Tabares et al. (2018). The light cycle consisted of 10 h of light and 10 h of darkness, in between 2 h for dawn and 2 h for dusk. On the ground of plastic boxes for gastropod maintenance, a layer of coconut soil (Kokosfaser–Humusziegel, TropicShop, Germany) of approximately 5 cm thickness was used. Exclusively parasite-free newly bred *L. fulica* (i.e., F1 and F2 generations) were used for *A. vasorum* infections. These F1/F2 *L. fulica* were fed with lettuce (*Lactuca sativa*), carrots (*Daucus carota*), cucumber (*Cucumis sativus*), zucchini (*Cucurbita pepo*), and commercial dog food (Premium Trockenfutter Romeo, Germany). In order to raise the pH of the soil, garden lime was mixed into the coconut soil. Furthermore, common cuttlefish (*Sepia officinalis*) internal shells (Sepiaschalen, TropicShop, Germany) were offered ad libitum to *L. fulica* as a natural source of calcium.

### 2.2. Isolation of Vital Angiostronglyus Vasorum L1 and Gastropod Oral Infection

Vital first-stage larvae (L1) were isolated from feces of naturally *A. vasorum*-infected dogs (kindly provided by the Institute of Parasitology at the University of Veterinary Medicine Hannover, Hannover, Germany) via the modified Baermann funnel technique, according to Conboy et al. [50]. After 24 h of incubation, 7 mL were obtained from each funnel apparatus, quantified microscopically (Olympus BH-2), and pelleted (200× *g*, 5 min) to collect vital *A. vasorum*-L1. *L. fulica* (*n* = 26) were orally infected with a dose of 1000 larvae each. In order to be as close as possible to field conditions, the following procedure was used for infection: since gastropods are coprophagic, the remaining fecal solution from the Baermann funnels was used as a suspension medium for the larvae. Thus, 50 mL of diluted canine fecal solution (DCFS) containing 1000 motile larvae were orally fed to the snails via a syringe until complete consumption (please refer to Video 1).

### 2.3. Gastropod Euthanasia and Organ Isolation

*A. vasorum*-infected *L. fulica* snails were cryo-euthanized in an ice bath at 4 °C, as reported previously by Lange et al. [51], at different time points post infection (p.i.; please refer to Supplementary Table S1). Following euthanasia, the snails were dissected, and the following 11 organs/tissues were isolated: crop/esophagus, stomach, intestine, hepatopancreas, reproductive tract, albumen gland, foot, kidney, lung, head, and heart. Four gastropods dissected at 5, 14, 29, and 47 days p.i. were used for histological analyses on the different tissues and organs. The organs of these four snails were fixed in 10% neutral-buffered formalin for further histological analyses.

### 2.4. Artificial Digestion and Identification of Larvae

A total of 22 of the dissected snails were further processed by artificial digestion. Snail single organs and tissues were artificially digested, according to Lange et al. [51]. Afterward, the remains were microscopically analyzed applying 40×, 200×, and/or 400× magnification (Olympus BH-2). To differentiate *A. vasorum* larvae (e.g., L1, L2, L3) from free-living or parasitic larvae of gastropods (genus *Phasmarhaptidis*), the type and length of the esophagus (e.g., non-rhabditiform, 1/2–1/3 of total larvae length), the tail form, and the length and width of the larvae were analyzed [52,53].

### 2.5. Histology

In total, 11 organs and tissues of four euthanized snails were used for histological analysis. As a first step, the snail’s shell was carefully removed. Thereafter, the inner organs were separated and fixed in 10% buffered formalin and embedded in paraffin wax by standard techniques. Tissue/organ sections of 5 µm thickness were cut from paraffin-embedded specimens and stained following the standard hematoxylin and eosin (HE) protocol for histological examination. HE staining was performed at the Institute of Pathology at the Faculty of Veterinary Medicine, Justus Liebig University Giessen, Giessen, Germany. The tissue slides were analyzed using an optical light microscope (Olympus BH-2) equipped with a digital camera (Olympus SC30) at the Institute of Parasitology of the Justus Liebig University Giessen, Germany.

## 3. Results

### 3.1. Angiostrongylus Vasorum Larval Development and Organ Tropism in Experimentally Infected L. fulica Snails

In order to investigate in detail *A. vasorum* larval organ tropism and development in vivo, single organs of *L. fulica*-infected snails were analyzed for the presence of different larval developmental stages. Overall, all 26 experimentally infected *L. fulica* proved positive for *A. vasorum* larvae. Even though all snails had obtained the same infection dose (1000 L1), different larval recovery rates per animal were observed when summing up all larvae found in one specimen. Thus, the overall larval recovery rates ranged from 1.8% to 49.7% (Table 1).

**Figure 1:**
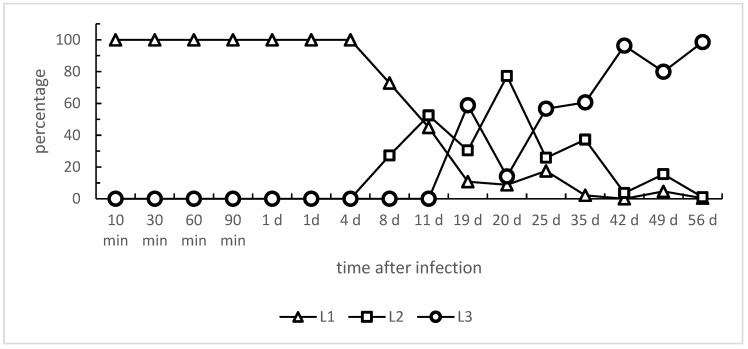
*Angiostrongylus vasorum* larval development in experimentally infected *Lissachatina fulica.*

Referring to the larval development within *L. fulica* snails, L1 stages started to disappear with day 8 p.i. ongoing; in parallel, L2 stages were first detected at 8 days p.i. (Figure 1). The proportion of recovered L2 stages peaked at day 20 p.i. and thereafter declined until the end of the investigation period (56 days p.i.). The first L3 stages were found at 11 days p.i., and the proportion of L3 in snails increased from this time point onwards; consequently, at day 56 p.i., most of the larval stages had developed into L3 (see Figure 1). Of note is that the speed of larval development varied between individual *L. fulica*. Hence, even after 49 days p.i., some larvae had not developed into the infective L3 stage (please refer to Figure 1 and Table 1). However, when analyzing *L. fulica* long-term infections of two years p.i., vital *A. vasorum* L3 were still found in all four cryo-euthanized snails, even though recovery rates had considerably dropped to 0.1–4.4% after this long period (Table 2).

**Figure 2:**
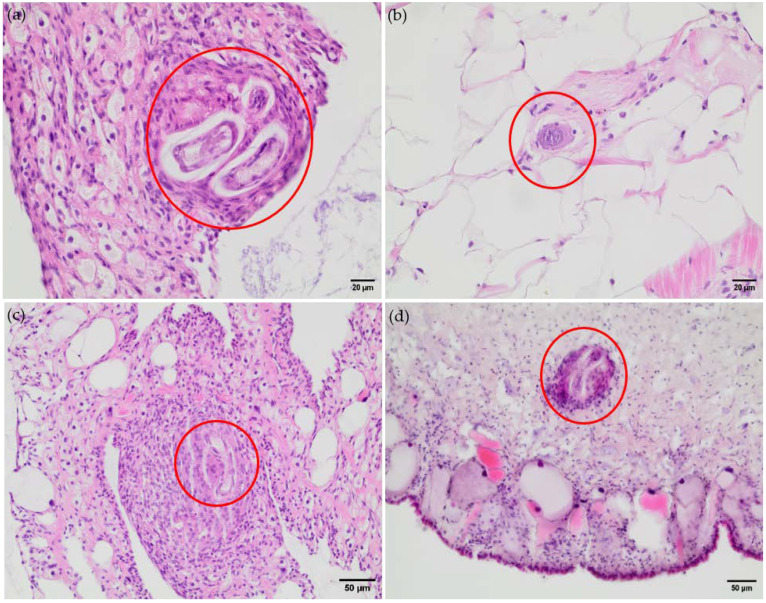
*Angiostrongylus vasorum* larvae in snail tissues (indicated by red circles), HE stained, 40x. (**a**,**b**): lung, 29 days p.i.; (**c**): lung, 5 days p.i.; (**d**): foot, 47 days p.i.

Analyses on parasite dispersion in *L. fulica* snails indeed revealed a marked tropism of *A. vasorum* larvae for specific organs and tissues, dependent on the time point of infection. Thus, the overall majority of larvae were found in lung and foot (muscles) tissues, while other organs such as the head, heart, albumen gland, crop-esophagus, intestine, kidney, and reproductive tract contained only a few larval specimens (Table 1). Referring to larval lung tropism, an interesting finding was observed here. The lungs of one *A. vasorum*-infected snail showed macroscopically visible, multiple calcified granulomas, most likely reflecting effective hemocyte-mediated innate immune reactions against invasive *A. vasorum* larvae (Figure 2).

Of note, *A. vasorum* L1 stages rapidly migrated in experimentally infected *L. fulica*, reaching distant organs already within 10 min after oral infection. In addition, the overall L1 migration highly differed between individual snails. As such, at 10 min p.i., 28% and 21% of L1 larvae were still located in the esophagus and stomach, respectively, while 24% were already found in the foot and 13% in the head. At 30 min p.i., most L1 larvae were localized in the foot (50%); 18.75% were found in the head and in the lungs. At 60 min p.i., a larger proportion of L1 was located in the hepatopancreas (36.07%), in the foot (22.4%), and in the head (15.85%). At 90 min p.i., 32%, 25%, and 12% L1 were located in the foot, head, and hepatopancreas, respectively. In one *A. vasorum*-infected snail, most L1 was detected in the foot (60.42%) at 1 day p.i., while only 19.72% of L1 larvae were located in the foot in another snail harboring 49.30% in the lung instead. Four days p.i., most larvae were observed in the lung (39.02%). Nonetheless, the head (17.07%), foot, and hepatopancreas (both with 12.20%) also contained relatively high proportions. At later time points, e.g., 8 days p.i., a high proportion of L1 was found in the hepatopancreas (41.77%), lung (27.85%), and stomach (17.72%). For the first time, L2 larvae were observed, but without any preference for specific organs. At 11 days p.i., most larvae were located in the lung (36.51%) and foot (17.06%). On this day, the first L3 larval stage was observed, being located in the heart. Of note, two-thirds of the observed larvae were still in the L2 stage. At 19 days p.i., 43.14% and 27.45% of the larvae were located in the lung and hepatopancreas, respectively. It can be noted that in the lung, the vast majority of larvae were L3 stages (40/44), while most larvae in the hepatopancreas were still L2 stages (18/28). At 20 days p.i., more than half of the larvae were found in the lung (55.26%), besides hepatopancreas (21.93%). In line, a high proportion of larvae was observed in the lung (34.09%) and foot (32.18%) at 25 days p.i. Here, the majority of larvae (86/136) had developed into L3 stages. Equally, at 35 days p.i., the lung (46.76%) and foot (29.90%) harbored most of the larvae, with more than half of the larvae (59.76%, 153/256) representing L3 stages. At 42 days p.i., larvae were mainly found in the lung (33.88%), hepatopancreas (17.13%), and stomach (17.13%). L1 larval stages were no longer observed, and almost all larvae were in the L3 stage (97.8%; 489/500). At 49 and 56 days p.i., mainly L3 stages (98.9%) were located in the lung (36.85% and 47.76%, respectively) and foot (45.70% and 25.52%, respectively).

**Figure 3:**
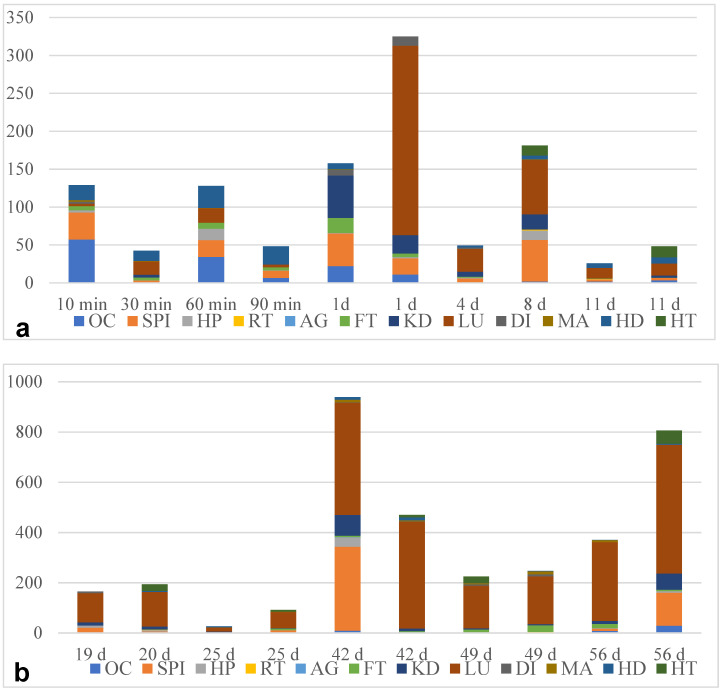
Larval burden of *Angiostrongylus vasorum* in *Lissachatina fulica* tissue in larvae per g organ weight. OC: esophagus and crop, SPI: stomach and proximal intestine, HP: hepatopancreas, RT: reproduction tract, AG: albumen gland, FT: foot, KD: kidney, LU: lung, DI: distal intestine, MA: mantle, HD: head, HT: heart. (**a**) <14 d p.i.; (**b**) >14 d p.i.

Considering larval burdens, clear differences were stated for the different organs (Figure 3). In general, the lung showed a high larval burden [up to 511 larvae per gram (L/g) of organ tissue] and, therefore, was the most infected organ, especially at days 42, 49, and 56 p.i. In contrast to this finding, the reproduction tract and albumin gland showed much lower larval burdens (0.83–1.75 L/g and 2.45 L/g, respectively). Of note, the different organs showed high interindividual variations in terms of larval burden (Table 2). Overall, the larval burden did not affect the larval developmental speed.

### 3.2. Histological Findings Indicate Hemocyte Responses to Angiostrongylus Vasorum Larval Stages

The analysis of histological samples confirmed the presence of *A. vasorum*-larvae in snail tissue, e.g., organs like lungs and feet (Figure 2). In the snail’s lungs, parasites were found separated by thin hemocyte-derived layers localized in the connective tissue surrounding blood vessels. As an exemplary finding in the connective tissue, we detected a single capsule enclosing three larvae at a time, while other larvae were found separated by thin hemocyte-derived layers (Figure 2). Due to the lack of karyolysis and/or foamy cytoplasm of these gastropod phagocytes, a clear hemocyte activation could not be confirmed for this microenvironment. Moreover, the larvae seemed still intact and showed no signs of cuticle destruction. In the thin gas exchange surface, no hemocyte accumulation was found in close vicinity to *A. vasorum* larvae, but a thin layer of connective tissue surrounded these pulmonary stages. In the snail foot, many *A. vasorum* larvae were located in the connective tissue. Numerous hemocytes accumulated around these parasitic stages, thereby forming a multicellular capsule. Herein, karyolysis was observed in some of these phagocytes, indicating hemocyte activation, while other hemocytes surrounding the larvae appeared to be flattened and potentially not activated.

**Figure 4:**
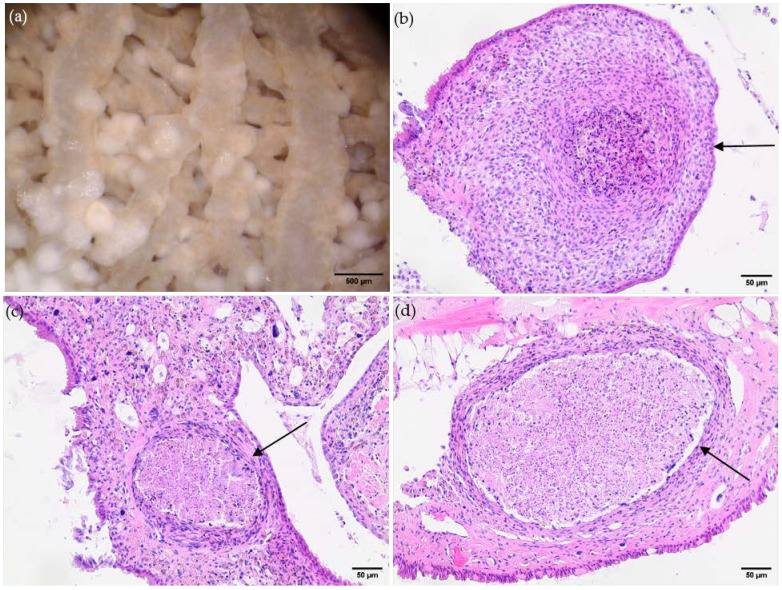
Granuloma-like structures in lung tissue of a long-term infected *Angiostrongylus vasorum*-infected African giant snail (*Lissachatina fulica*). (**a**): macroscopical illustration of the snail’s lung; (**b**–**d**): microscopical illustration of granuloma-like structures (indicated by black arrow).

As an interesting finding, the macroscopical analysis of the lungs of a long-term infected *L. fulica* snail (euthanized two years p.i.) revealed the presence of multiple calcified granulomas (Figure 4). These pathologic structures were excised, stained, and examined by bright field microscopy. Here, the classical characteristics of granuloma were confirmed histologically, but no larvae were found in any of these pulmonary hemocyte-derived structures, most probably indicating successful larval degradation.

## 4. Discussion

Detailed investigations on *A. vasorum* larval migration, organ tropism, development, and hemocyte-derived innate immune reactions within gastropod intermediate hosts are still very limited [40,54,55,56]. The present study confirmed *L. fulica* as a suitable obligate intermediate host for *A. vasorum*, as previously reported in tropical biomes of Colombia [28]. Consequently, all *A. vasorum* developmental larval stages were found in the host, L1, L2, and infective L3. Interestingly, in some experimentally infected *L. fulica*, vital *A. vasorum* L3 was still present two years after infection, indicating long-term larval survival in these hosts. These long-living stages might be part of not only parasitic survival strategies apart from the canid definitive host but also be relevant for parasite dissemination and transmission. Of note, the neozoan *L. fulica* can live for up to 9 years and thereby migrate over longer distances when compared to short-living terrestrial gastropods [28,29]. As such, *L. fulica* has demonstrated an alarming capacity for rapid spread. In the US State of Florida, three abandoned snails led to massive multiplication and the discovery of more than 18,000 individuals only seven years later, and currently, 15 US states have been colonized by this neozoan species [57]. Even though the mechanisms of *A. vasorum* spreading into non-endemic areas are still under debate [55,56], geographic shifts in mollusc intermediate host populations are assumed as one underlying factor [58,59]. Meta-analyses have revealed that both global warming and anthropogenic pressure strongly affect the phenology of terrestrial gastropods, including slugs, semi-slugs, and snails [16,60].

Referring to experimental infections, a high variation in *A. vasorum* recovery rates was rather obvious in the current study, even though applying the same infection dose (1000 L1/snail). A reason for these deviations might be seen in individual innate immune reactions mediated by hemocytes, as already reported for *Arion* spp. slugs and *L. fulica* snails [30,38,52]. As recently demonstrated in vitro, exposed hemocytes not only firmly adhered to the cuticle of *A. vasorum* L1 and L3 but also reacted by forming invertebrate extracellular phagocyte traps [40,54]. In line, hemocytes circulating within the hemolymph system are considered multifunctional professional phagocytes in gastropods, thereby resembling leukocytes of vertebrates [39,40,61,62,63]. Hemocytes display diverse effector mechanisms against invasive pathogens, including phagocytosis, encapsulation, cell-mediated cytotoxicity, and release of invertebrate extracellular phagocyte traps (InEPTs) [40,42,54,64]. Some of these hemocyte-derived defense mechanisms were here confirmed in histological analyses of *A. vasorum*-infected *L. fulica* tissues. Hence, accumulation of hemocytes around larvae was found in addition to multicellular larval encapsulation, leading to the formation of large granuloma. Importantly, *A. vasorum* larvae, being surrounded by hemocytes, were detected in several tissues like the foot, distal intestine, lung, and the mantle of snails as early as 10 min p.i. These findings indicated that despite a fast *A. vasorum* larval migration, hemocytes are rapidly recruited to sites of parasite infection [40,54]. Rapid hemocyte recruitment might be a consequence of the open circulatory system of gastropods, with the hemolymph flowing through sinuses and thereby directly bathing organs. Thus, inner organs are surrounded by hemolymph containing circulating hemocytes, which may immediately react against pathogens, including *A. vasorum* larvae [65,66]. Interestingly, percutaneous *A. vasorum* L1 infection of molluscs resulted in InEPTs within gastropod mucous extrapallial space in vivo [30]. Accordingly, gastropod hemocytes spontaneously reacted against vital *A. vasorum* L1 within 30 min by forming InEPTs, resulting in firm entrapment of these motile larvae [54].

In the current study, *A. vasorum* L2 was found as early as 8 days p.i., and the first fully developed L3 stages were present at 11 days p.i. in snail tissues. Consequently, canids might become infected via *L. fulica*-derived L3 as early as 11 days p.i. Compared to a previous report stating a duration of 17 days for *A. vasorum* L3 development [36], the current data indicated a faster development using the same gastropod intermediate host species, which may be due to the climate chamber-based standardized experimental conditions (i.e., a circadian cycle of 10 h of light, 10 h of darkness, 20–26 °C, and 50% humidity) used in the current study. As well documented for other intermediate host species like mosquitoes [67,68], the larval development within intermediate hosts strongly depends on climatic conditions. In line with current observations, the individual developmental rate varied significantly under natural conditions [36]. Hence, the first L3 stages were detected at 11 days p.i., but some L1 stages were still found in foot tissues at 56 days p.i. in a few snail individuals, thereby verifying an asynchronous development of *A. vasorum*-larvae in intermediate hosts. This phenomenon might rely on different biological aspects, such as varying oxygen concentrations and biochemical, cellular, and immunological conditions in different organs or within the same organ, finally resulting in delayed individual larval development. Alternatively, a discontinued or asynchronous development could represent a kind of larval hypobiosis known from numerous other parasitic nematodes [69,70] to overcome adverse exogenous or even endogenous in vivo conditions.

Regarding organ tropism, clear differences were observed in the early (<1 d) and late (>1 d) stages of infection. Hence, the esophagus and stomach showed a higher larval concentration shortly after an oral infection due to the passive passage of the liquid-containing larvae through the proximal digestive tract. In the hepatopancreas, larval proportions strongly fluctuated, ranging from 0% (11 days p.i.) to 41.77% (8 days p.i.). The physiological functions of the hepatopancreas include resorption and storage of nutrients, as well as mucus and enzyme production [71], thereby representing a potential nutrient source for larval development. In addition, this organ also owns an immune function [71,72], which plays a role in fighting invading pathogens, eventually explaining the varying numbers of larvae in this organ.

Of note, several organs, such as the heart, mantle, and distal intestine, showed a low larval presence throughout the entire infection period. This may be linked to the fact that these inner organs have fewer metabolic activities and nutrient abundance, thus being less favorable for larval development. Conversely, a high larval concentration was observed in the lung in later phases of infection (up to 72.65%) as opposed to earlier time points (max. 18.75%). Here, anatomical restrictions may hamper the larvae from directly migrating to this location, most likely due to the passive flow of the digestive tract, the anatomical location of the lung, and the breathing mechanism [36].

Obviously, the number of *A. vasorum* larvae present in these tissues reflects the organ tropism; however, favorable organ conditions may also be linked to accelerated larval development into L3 stages. As specifically detected on days 20, 25, and 49, the relation between the L2 and L3 stages balanced in favor of the L3 stages in the lung when compared to the foot or hepatopancreas. An accelerated development might be associated with favorable organ-specific metabolic activities or high-rate hemolymph circulation, thereby delivering a more effective nutrient supply to fulfill the needs of *A. vasorum* larvae. The same holds true for gas exchange, including CO_2_ and O_2_, as previously postulated elsewhere [36]. As reported for other closely related metastrongyloid lungworm species, *A. vasorum* seems to favor hyperoxic conditions (like in the snail lung) since not only the late larval but also adult stages are allocated in the right heart and *Arteria pulmonalis* in the canid definitive host [20,73]. In contrast to lung tissues, very few *A. vasorum* larvae were found in the reproductive tract of hermaphrodite snails, consisting of the uterus, oviduct, vas deferens, penis, vagina, ovotestis and gonopore [36,74], but also of the albumen gland, which secretes the perivitelline fluid being deposited on the eggs of the snail. Both the gland’s size and activity are closely linked to the sexual cycle of snails, therefore not representing an ideal place for lungworm larval development. In agreement with current findings, a minor role of the *L. fulica* reproductive tract for larval migration was also described by Sauerländer et al. [36]. However, in contrast to *A. vasorum*, the gastropod reproductive tract may represent a target organ for other parasites, such as trematodes [71,75].

As expected, histological analyses of infected snail tissues confirmed that *L. fulica* indeed mounts an innate immune response against *A. vasorum* larvae. Consistently, the strongest hemocyte-mediated reactions were found in foot and lung tissues. In the latter, hemocytes effectively encapsulated *A. vasorum* larvae by pulmonary granuloma formation. So far, the current data do not allow any conclusion on whether encapsulated larvae were killed since their cuticle still seemed intact. Besides granuloma formation, the first in vivo evidence of InEPT extrusion by activated hemocytes was found since some hemocytes showed karyolysis and a foamy cytoplasm, i.e., cellular events being associated with InEPT formation in vitro [40,54]. In contrast to the above findings, *A. vasorum* larvae present in the thin gas exchange surface neither induced hemocyte accumulation nor granuloma formation, indicating niche-specific reactions. However, we can only speculate that this may result from a general lower abundance of hemocytes in gas exchange areas to guarantee undisturbed gas exchange.

A striking evidence of granuloma formation was found in long-term *A. vasorum*-infected *L. fulica*, i.e., as late as two years after infection. Here, a massive accumulation of macroscopically visible granuloma was found in the lungs, unveiling marked snail-derived encapsulation of migrating larvae, as previously reported [30,45]. Histological analyses of these pathological structures confirmed the classical characteristics of granuloma. Surprisingly, neither fragments nor complete *A. vasorum* larvae were found inside these cellular structures. Thus, they may represent long-term remnants of effective hemocyte-mediated innate immune reactions, finally leading to effective killing and dissolvement of larvae. Prominent calcification processes in these granulomas may underline this assumption. Another interesting finding in one of these long-term-infected snails was the presence of vital larvae, primarily in the hepatopancreas. It is tempting to hypothesize that the hepatopancreas serves as a safe haven for L3 long-term survival, eventually based on a low abundance of hemocytes in this digestive gland. Undoubtedly, this hypothesis awaits further experimentation.

In conclusion, our findings confirm *L. fulica* as a suitable intermediate host for *A. vasorum* and further demonstrate the prolonged survival of infective L3 stages. Given that *L. fulica* can reach a remarkable age of nine years [72], this long-term survival of *A. vasorum* in its intermediate host may, on the one hand, aid parasite geographic spread to formerly free areas and, on the other hand, pose a considerable long-term risk to susceptible definitive hosts in several regions, endemic for this invasive snail species, such as Colombia, Ecuador, Cuba, the USA, Indonesia, or India [17,28,33,34,76,77]. The preference of *A. vasorum* larvae for certain organs indicates a complex interaction between the parasite and its intermediate host, which warrants further investigation.

## Figures and Tables

**Table 1:** Larval stages of *Angiostrongylus vasorum* in *Lissachatina fulica* tissue.

	OC	SPI	HP	RT	AG	FT	KD	LU	DI	MA	HD	HT	RR
10 min	L1	L1	L1	n.l.d.	n.l.d.	L1	n.l.d.	L1	L1	L1	L1	n.l.d.	10
30 min	L1	L1	L1	n.l.d.	n.l.d.	L1	L1	L1	n.l.d.	L1	L1	n.l.d.	4.8
60 min	L1	L1	L1	n.l.d.	n.l.d.	L1	n.l.d.	L1	n.l.d.	L1	L1	n.l.d.	18.3
90 min	L1	L1	L1	n.l.d.	n.l.d.	L1	n.l.d.	L1	n.l.d.	n.l.d.	L1	n.l.d.	8.8
1 day	L1	L1	L1	n.l.d.	n.l.d.	L1	L1	n.l.d.	L1	L1	L1	n.l.d.	9.6
1 day	L1	L1	L1	n.l.d.	n.l.d.	L1	L1	L1	L1	n.l.d.	n.l.d.	n.l.d.	7.1
4 days	n.l.d.	L1	L1	n.l.d.	n.l.d.	L1	L1	L1	L1	n.l.d.	L1	n.l.d.	4.1
8 days	L1	L1, L2	L1, L2	L2	n.l.d.	n.l.d.	L1, L2	L1, L2	n.l.d.	L1	L1, L2	L1	15.8
11 days	L1	L2	L1	L1	n.l.d.	L1, L2	n.l.d.	L2	n.l.d.	n.l.d.	L1	n.l.d.	2.1
11 days	L2	L2	n.l.d.	n.l.d.	n.l.d.	L1	L2	L2	n.l.d.	n.l.d.	L1, L2	L3	1.8
19 days	L3	L1, L2, L3	L1, L2, L3	n.l.d.	n.l.d.	L1, L2, L3	L1, L2, L3	L2, L3	L2	n.l.d.	n.l.d.	n.l.d.	10.2
20 days	n.l.d.	L2, L3	L1, L2, L3	L2	n.l.d.	L1, L2	L2	L2, L3	n.l.d.	L2	L1, L2	L2, L3	11.4
25 days	n.l.d.	L2, L3	n.l.d.	n.l.d.	n.l.d.	L1, L2, L3	L2, L3	L3	n.l.d.	L3	L1, L2, L3	n.l.d.	2.7
25 days	n.l.d.	L2, L3	L1, L2	n.l.d.	n.l.d.	L1, L2, L3	L3	L3	n.l.d.	L3	n.l.d.	L3	10.9
35 days	n.l.d.	L2, L3	L2, L3	L2, L3	n.l.d.	L2, L3	n.l.d.	L2, L3	n.l.d.	L2, L3	L2	L2, L3	11.6
35 days	n.l.d.	n.l.d.	L2, L3	L2	n.l.d.	L1, L2, L3	L1, L2, L3	L2, L3	L3	L3	L2, L3	L2, L3	13.9
42 days	L3	L3	L2, L3	L3	L3	L2, L3	L3	L3	L3	L3	L3	n.l.d.	40
42 days	n.l.d.	n.l.d.	L2, L3	L3	n.l.d.	L2, L3	L3	L3	L3	L3	L2, L3	L3	10
49 days	L3	L3	L3	L1	n.l.d.	L1, L2, L3	L1, L3	L1, L3	L3	L2, L3	L1	L3	14.2
49 days	n.l.d.	n.l.d.	L1, L2, L3	L3	n.l.d.	L1, L2, L3	L3	L2, L3	L3	L2, L3	L2, L3	n.l.d.	29.6
56 days	L3	L3	L3	n.l.d.	n.l.d.	L1, L2, L3	L3	L3	n.l.d.	L3	L3		26.9
56 days	L2, L3	L3	L3	n.l.d.	n.l.d.	L3	L3	L3	L3	L3	L3	L3	49.7

OC: esophagus and crop, SPI: stomach and proximal intestine, HP: hepatopancreas, RT: reproduction tract, AG: albumen gland, FT: foot, KD: kidney, LU: lung, DI: distal intestine, MA: mantle, HD: head, HT: heart, RR: recovery rate in %. Color coding: number of larvae found in the organs: red > 20, pink: 5–20, light pink: < 5. n.l.d.: no larvae detected.

**Table 2:** Larval stages of *A. vasorum* in *Lissachatina fulica* tissues after long-term infection (2 years).

	MA, HT	RT	AG	HD	KD	FT	OC	HP	ST	LU	IR
2 y	L3					L3				L3	0.8
2 y					L3	L3					0.2
2 y			L3			L3	L3	L3	L3	L3	4.4
2 y						L3					0.1

MA, HT: mantle and heart, RT: reproduction tract, AG: albumen gland, HD: head, KD: kidney, FT: foot, OC: esophagus and crop, HP: hepatopancreas, ST: stomach, LU: lung, IR: infection rate in %, Color coding: number of larvae found in the organs: red > 20, pink: 5–20, light pink: < 5. n.l.d.: no larvae detected.

## Data Availability

Data are contained in this article.

## Supplementary Materials

The following supporting information can be downloaded at: https://www.mdpi.com/article/10.3390/pathogens13110946/s1.
