# Supplementary material for: Organ Tropism of Angiostrongylus vasorum Larval Stages in Infected African Giant Snails (Lissachatina fulica)
Source: Pathogens. 2024 Oct 30;13(11):946. doi: 10.3390/pathogens13110946 (PMC11597215; doi:10.3390/pathogens13110946)
Supplement: Supplementary file 1 [file pathogens-13-00946-s001.zip › Supplementary materials.pdf]

Supplementary materials

Time points after *A. vasorum*-infection	10 min	30 min	60 min	90 min	1 day	4 days	8 days	11 days	19 days	20 days	25 days	35 days	42 days	49 days	56 days	2 years
Number of snails	1	1	1	1	2	1	1	2	1	1	2	2	2	2	2	4

Supplementary Table S1. Euthanasia time points after *Angiostrongylus vasorum* infection and number of euthanized *L. fulica*.

video oral infection
achatina fulica _Trim

Video S1: Oral infection of *L. fulica* snails with *A. vasorum* L1 larvae
